# An epidemiological investigation of leukemia incidence between 2003 and 2007 in Nanjing, China

**DOI:** 10.1186/1756-8722-3-21

**Published:** 2010-06-02

**Authors:** Bao-An Chen, Zhi-Hu Huang, Xiao-Ping Zhang, Jian Ou-Yang, Jian-Yong Li, Yong-Ping Zhai, Xue-Mei Sun, Yan-Li Xu, Qin Lu, Jian-Min Wang, Dong Li, Hui Liao, Zhi-Xiang Shen, Yan-Yan Wang, Xiao-Jing Yu, Hui Ye, Li-Ying Zhuang

**Affiliations:** 1 Department of Hematology, Zhongda Hospital Affiliated to Southeast University, Nanjing, PR China; 2 Depqrtment of Hematology, The Affiliated Drum Tower Hospital, Medical School of Nanjing University, Nanjing, PR China; 3 Department of Hematology, The First Hospital Affiliated to Nanjing Medical University, Nanjing, PR China; 4 Department of Hematology, Nanjing General Hospital of Nanjing Military Command, Nanjing, PR China; 5 Department of Hematology, Jiangsu Provincial Hospital of Traditional Chinese Medicine, Nanjing, PR China; 6 Department of Hematology, Nanjing First Hospital, Nanjing, PR China; 7 Department of Hematology, Nanjing Children's Hospital, Nanjing, PR China; 8 Department of Hematology, The Second Hospital Affiliated to Nanjing Medical University, Nanjing, PR China; 9 Department of Hematology, Hospital NO 454 P.L.A, Nanjing, PR China; 10 Department of Hematology, Jiangbei People's Hospital, Nanjing, PR China; 11 Department of Hematology, Ruijin Hospital Affiliated to Shanghai Jiao Tong University, school of medicine, Shanghai, PR China; 12 Public Health school, Southeast University, Nanjing, PR China; 13 School of Clinical Medicine, Southeast University, Nanjing, PR China; 14 Group of Hematology in Nanjing (GHN

## Abstract

**Background:**

There has been little literature about leukemia epidemiology in Nanjing in recent years. We aimed to explore the incidence rate, gender and age distribution of leukemia in Nanjing using the leukemia database of the city.

**Results:**

The average yearly incidence rate of leukemia was 3.68/10^5 ^during 2003 - 2007 in Nanjing. There were no significant difference in gender (*x*^2 ^= 3.266, *p *> 0.05) or seasons (*x*^2 ^= 11.36, *p *> 0.05). The incidence rate was the highest in group aged 80~ years (18.64/10^5^). AML accounted for approximately 36.8% of all leukemias.

**Conclusions:**

The incidence rate of leukemia, especially in the aged population, was relatively high in Nanjing. Leukemia is the major malignant tumor in children. Therefore, more attention should be paid to leukemia in children and the aged people.

## To the Editor

Nanjing, an ancient capital and the second largest city in southeast China, enjoys a worldwide reputation for its long history and splendid culture. Little has been reported regarding leukemia epidemiology of the city in recent years. The present study was to investigate the leukemia incidence rate in Nanjing during 2003-2007, which may be of help to further leukemia etiology research.

The leukemia data were collected from all hospitals which are capable of making a definite diagnosis of leukemia. We diagnosed leukemia by blood test, bone marrow puncture, histochemical staining and typing and classified by the "French American British" classification. The population data were provided by Nanjing Public Security Bureau and Nanjing Statistical Yearbook in 2007. There were 1,095 cases of various types of leukemia, while the total population in Nanjing during 2003-2007 was 29.7603 million, so the overall average annual incidence rate of leukemia was 3.68/10^5 ^and the age-standardized incidence rate was 3.47/10^5^. The result was higher than that of the entire nation (2.76/10^5^) [1], but lower than that in some other cities in China, such as: Shenyang(4.83/10^5^) [2] and Tianjin during 1981-2000(4.71/10^5^) [3]. Moreover, compared with the whites' leukemia incidence rate (12.8/10^5^), it was lower.

Table 1 shows incidence rates of all types of leukemia in every age group in Nanjing during 2003 to 2007. The leukemia incidence rate in old age groups was generally high, and reached to the peak in group aged 80~ years(18.64/10^5^), but this was still lower than the highest rate in Shenyang-the group aged 75~ years (35.76/10^5^) [2]. Although the incidence rate of leukemia in children was lower than that in the old age group, leukemia accounted for a big proportion in the child malignant tumors [2,4], therefore, leukemia has become the children's primary malignant tumor. The possibe explanation was bad internal environment's stable of hemopoiesis system in children, the hereditary factors, prenatal exposure to hazard factors of leukemia (X-ray, diesel oil, chemical fertilizer) and so on [5]. Other research presumed childhood leukemia was related with poor immunity and high birth weight [6].

**Table 1:** Leukemia subtype incidence between 2003 to 2007 for different age groups in Nanjing, China

Age (year)	Rate(1/10^5^)						
	
	AML	ALL	AUL	CLL	CML	Other	Total
0~	0.49	2.95	0.49	0	0	0	3.93
1~	0.23	3.60	0.46	0	0.12	0	4.41
5~	0.48	1.51	0.14	0.07	0.14	0	2.34
10~	0.77	0.68	0.14	0.1	0	0.05	1.74
15~	0.43	1.06	0.09	0	0.26	0.04	1.87
20~	1.15	0.92	0.15	0.04	0.42	0.15	2.85
25~	0.75	0.37	0	0.04	0.45	0	1.61
30~	0.56	0.41	0.09	0.03	0.38	0.03	1.50
35~	0.98	0.26	0.2	0.1	0.85	0.07	2.46
40~	1.42	0.49	0.49	0.31	1.29	0.04	4.03
45~	1.50	0.63	0.17	0.17	0.75	0.04	3.25
50~	2.06	0.89	0.33	0.67	1.17	0.17	5.29
55~	1.53	1.05	0.32	0.97	1.53	0.32	5.73
60~	3.18	0.73	0.64	0.54	1.54	0.09	6.72
65~	3.25	1.12	0.91	2.23	0.91	0.30	8.73
70~	4.83	0.94	0.54	1.61	1.61	0.67	10.20
75~	4.68	1.49	1.28	4.68	2.34	0.43	14.88
80~	7.99	0	0.44	6.21	3.11	0.88	18.64
85~	2.55	1.27	2.55	3.82	3.82	1.27	15.29
Total	1.35	0.81	0.27	0.41	0.73	0.11	3.68
composition ratio(%)	36.8	22.1	7.3	19.7	11.2	2.9	

Interestingly, AML accounted for approximately 25% of all leukemias in adults and 15%-20% in patients age ≤ 15 years in the Western world[7], which were both much lower than 36.8% in our research. The incidence rates of all types of leukemia especially non-AML types were lower in Nanjing.

The composition ratio of different subtypes of AML were: M2(36.17%), M3(26.60%), M5(11.35%), M4(10.28%), M1(6.74%), M6(4.96%), M7(2.13%), M0(1.77%), respectively. These were similar to the reports in the literature [8,9].

By chi-square test, no significant difference was found between genders (*x*^2 ^= 3.266, *p *> 0.05) or among seasons (*x*^2 ^= 11.36, *p *> 0.05). And the 5-year incidence rate is relatviely stable.

Current study has provided detailed information of the leukemia epidemiology in Nanjing during the period 2003-2007. Long-term follow-up investigations are needed for better understanding of characteristics of leukemia in Nanjing, including etiology, survival and risk indicators, which may lend support to the corresponding protocols for prevention and intervention.

## Abbreviations

FAB: French-American-British Cooperation Group; ALL: acute lymphocytic leukaemia; AML: acute myeloid/myelogenous leukemia; CLL: chronic lymphocytic leukemia; CML: chronic myelocytic leukemia; AUL: acute unclassificatied leukemia.

## Competing interests

The authors declare that they have no competing interests.

## Authors' contributions

Bao-An Chen, Zhi-Hu Huang and Xiao-Ping Zhang collected the data and contributed to analysis and writing. Jian Ou-Yang, Jian-Yong Li, Yong-Ping Zhai, Xue-Mei Sun, Yan-Li Xu, Qin Lu, Jian-Min Wang, Dong Li, Hui Liao, Zhi-Xiang Shen, Yan-Yan Wang collected the data. Xiao-Jing Yu, Hui Ye, Li-Ying Zhuang did the statistical analyses.
